# Global Research Landscape on Plastic Microfibers in Sludge Treatment: Proteomic Mechanisms and Biotechnological Pathways for Biomass Valorization

**DOI:** 10.3390/polym18060734

**Published:** 2026-03-17

**Authors:** S. Jonathan Rojas-Flores, Rafael Liza, Renny Nazario-Naveda, Félix Díaz, Daniel Delfin-Narciso, Moisés Gallozzo Cardenas, Luis Cabanillas-Chirinos

**Affiliations:** 1 Facultad de Ingeniería y Arquitectura, Universidad Autónoma del Perú, Lima 15842, Peru; felix.diaz@autonoma.pe; 2 Escuela de Posgrado, Universidad Continental, Lima 15113, Peru; rlizan@continental.edu.pe; 3 Departamento de Ciencias, Universidad Tecnológica del Perú, Trujillo 13011, Peru; c30216@utp.edu.pe (R.N.-N.); c21228@utp.edu.pe (M.G.C.); 4 Grupo de Investigación en Ciencias Aplicadas y Nuevas Tecnologías, Universidad Privada del Norte, Trujillo 13011, Peru; daniel.delfin@upn.edu.pe; 5 Institutos y Centro de Investigación, Universidad Cesar Vallejo, Trujillo 13001, Peru; lcabanillas@ucv.edu.pe

**Keywords:** plastic microfibers, sludge dewatering, extracellular polymeric substances (EPSs), proteomics, biotechnology, biomass valorization

## Abstract

Plastic microfibers (PMFs) increasingly accumulate in wastewater treatment plants, impairing sludge dewatering and raising operational costs. This study combines a bibliometric analysis (2000–2025) with a critical review of the recent mechanistic literature to map the evolving research landscape on PMF–extracellular polymeric substance (EPS) interactions. The bibliometric trajectory (R^2^ = 0.9786) underscores a paradigm shift towards a molecular understanding of the sludge matrix. Our synthesis of recent experimental studies reveals that PMF-induced interference is often driven by the selective adsorption of hydrophobic extracellular proteins, with one study reporting up to 32.5% sequestration. This has been linked to deteriorated dewatering, such as a 45% increase in capillary suction time (CST) under controlled conditions. Proteomic studies have identified more than 40 extracellular proteins with altered expression, directly linking PMFs to impaired sludge rheology. However, this review critically assesses the underlying evidence, highlighting significant methodological heterogeneity, a lack of standardized protocols, and a reliance on laboratory-scale models as key limitations that prevent broad generalization. By identifying these gaps, this work reframes the PMF–EPS research agenda, emphasizing the need for harmonized methods and multi-omics integration to transform mechanistic insights into robust biotechnological solutions for sustainable sludge management within a circular bioeconomy.

## 1. Introduction

The growing presence of plastic microfibers (PMFs) in wastewater treatment systems constitutes an environmental and operational challenge [1]. Recent data quantify the magnitude of the problem: each cycle of washing synthetic clothing releases approximately 700,000 microfibers, of which up to 40% reach treatment plants [2]. In addition to release through abrasion during textile washing, recent research has revealed that plastics can discharge polymeric micropollutants into water via stress-induced phase separation mechanisms—a process that expands our understanding of the sources and dynamics of these contaminants in aquatic environments [1,2]. This translates into documented concentrations ranging from 1000 to 30,000 particles per liter in the influent of these facilities [3]. Monitoring studies in European and North American plants have shown that PMFs account for between 8% and 15% of total suspended solids in biological reactors, with documented cases where this fraction reaches 22% in plants serving areas with a high density of textile industries [4].

This massive accumulation has direct economic consequences: medium-sized treatment plants (capacity 100,000 m^3^/day) report increases of 20–35% in the consumption of flocculant polymers to compensate for impaired dewatering, resulting in additional costs that can exceed €150,000 annually per facility [5,6]. The presence of PMFs reduces dewatering efficiency by 18–42%, measured through capillary suction time (CST), and increases residual moisture in sludge cakes by 7–15 percentage points [7]. This interference compromises not only operational efficiency but also the economic viability of an essential process for water purification, in a context where sludge generation in the EU exceeds 10 million tons annually and management costs represent up to 50% of the total operating budget of a conventional plant [8].

This phenomenon highlights its growing scientific relevance and urgent translation into process engineering [9]. The state of the art has shifted from mere documentation of the problem toward elucidation of molecular mechanisms [10]. Statistically, the proportion of studies specifically analyzing PMF–EPS interactions grew from 12% in 2020 to more than 65% in 2024 [11,12,13]. Zhang et al. (2022) demonstrated that polyester microfibers adsorb up to 32.5% of hydrophobic EPS proteins, increasing CST by 45% and linking physical interaction for the first time to measurable deterioration in dewatering [14]. Sharma et al. (2024) applied proteomics, identifying 47 altered extracellular proteins (some overexpressed >400%) that explained a 60% increase in water retention and a 28% reduction in floc pore size [15]. The most relevant findings indicate that PMFs can selectively adsorb between 30% and 50% of key hydrophobic extracellular proteins, altering the architecture of the sludge matrix [15]. Finally, Müller et al. (2025) evaluated mitigation strategies, finding that enzymatic pretreatment reduced CST by 38% but increased ammonium in the center by 15%, revealing critical trade-offs [16].

The application of systematic bibliometric analysis, implemented with tools such as R Studio (via the Bibliometrix package, 5.2.1), VOSviewer (1.6.18), and Plotly Studio (3.3.0), enables an objective and in-depth exploration of the field [17]. This approach transcends traditional narrative reviews by offering a data-driven mapping. With R Studio, thousands of bibliographic records can be processed to identify precise temporal trends, author and institutional productivity, and collaboration patterns with reproducible metrics [18]. VOSviewer is employed to construct visual network maps that reveal the intellectual structure of the field: keyword clusters showing thematic cores (such as “EPS proteomics” or “sludge rheology”), co-citation networks identifying foundational articles and schools of thought, and collaboration networks highlighting the most active research consortia [19,20]. Meanwhile, Plotly Studio enhances the dissemination of findings by enabling interactive visualizations and dynamic dashboards, making complex patterns accessible and explorable [21]. Together, these tools not only describe the past and present of research but also project future trends and diagnose strengths and weaknesses in knowledge development, offering a strategic evidence-based roadmap [22]. Currently, the literature remains fragmented, lacking a visual and integrative synthesis that maps global knowledge of the PMF–proteome–EPS mechanism and clearly outlines future priority research directions. This absence of a unified framework limits coordinated progress toward applied solutions.

The main objective of this research is to conduct a comprehensive analysis of the state of scientific knowledge on the impact of plastic microfibers on the dewaterability of activated sludge, with particular emphasis on the mechanisms of proteome alteration and the structure of extracellular polymeric substances (EPSs), in order to identify trends, research cores, and future strategic directions. To this end, the following research questions will be addressed: Q1: What are the temporal trends and the most productive and cited authors, institutions, and journals in this research area (2015–2025)? Q2: How is the international collaboration network among researchers in this field structured, and how has it evolved? Q3: What are the main keywords and emerging research topics, and how have they been conceptually clustered and shifted over time? Q4: Which documents constitute the foundational literature (co-citation analysis), and which research lines have generated the greatest impact and influence? Q5: What are the main knowledge gaps and future priority research directions derived from the analysis of the existing scientific literature? It is important to emphasize that the behavior and impact of PMFs in sewage sludge differ significantly from other environmental compartments, such as seawater or soil. The activated sludge matrix is a complex and dynamic biological system, characterized by a high concentration of microbial biomass and a dense EPS network. Unlike aquatic environments, where PMFs primarily act as vectors of contaminants, in sludge, their physical and chemical presence directly interferes with floc structure. The high ionic strength and the presence of divalent cations (Ca^2+^, Mg^2+^)—which are crucial for EPS cross-linking and stability—can modulate PMF adsorption and their effect on dewaterability [13]. Therefore, interaction mechanisms and mitigation strategies must be studied specifically within this context and cannot be directly inferred from studies conducted in other media. This review focuses precisely on this niche.

## 2. Results and Analysis

**Figure 1:**
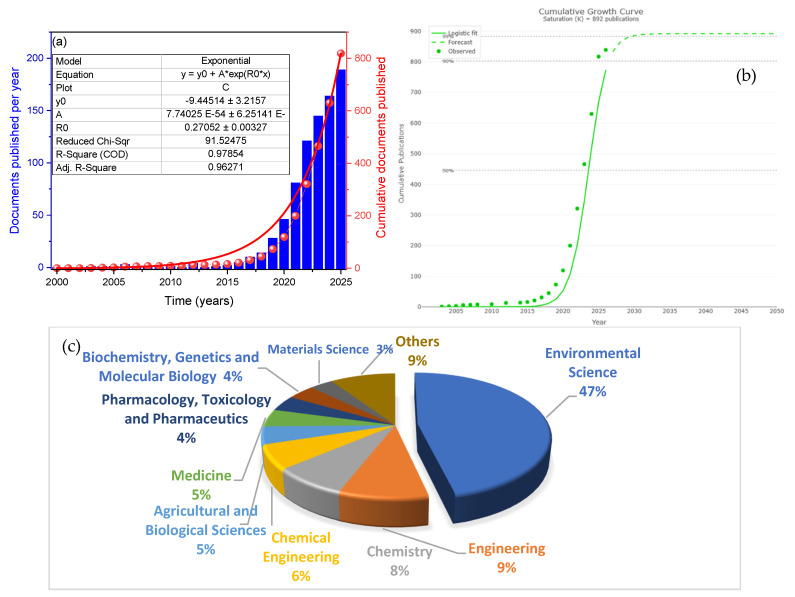
Comprehensive analysis of scientific productivity in the field, showing (**a**) cumulative exponential growth, (**b**) annual publication trends, and (**c**) disciplinary distribution.

In Figure 1a, the cumulative growth curve of publications from 2000 to 2025 is shown, fitted to an exponential model with the equation y = y0 + Aexp(R0x). The exponential model (R^2^ = 0.9786) confirms that the field is in a phase of rapid and sustained expansion, with no indication of saturation based on the data available up to 2025. The fitting parameters indicate accelerated and robust growth, with a coefficient of determination R^2^ = 0.9786 and an adjusted R^2^ = 0.9627, confirming that the trend is exponential rather than linear, with a growth rate of R_0_ = 0.2706. This reflects the rapid consolidation of the field, particularly after 2015, coinciding with growing global concern about microplastics. In Figure 1b, annual publication output is displayed, showing a marked and sustained increase beginning in 2018, with the highest annual counts observed in the most recent years of the analysis (2024–2025). This trend indicates that the topic has gained and continues to gain prominence in the international research agenda, with no evidence of a plateau or decline within the analyzed period. Finally, Figure 1c breaks down the disciplinary distribution of publications, revealing that 47% of the scientific output falls under environmental sciences, followed by engineering (9%), chemistry (8%), Chemical Engineering (6%), and other areas such as Agricultural and Biological Sciences (5%), Medicine (5%), and biochemistry, genetics, and molecular biology (4%). This distribution confirms the interdisciplinary nature of the field, though with a clear dominance of environmental sciences and engineering, underscoring its applied orientation toward water treatment and sludge management. Analytically, the exponential growth observed in Figure 1a not only quantifies the expansion of knowledge but also reflects a transition from early descriptive studies to mechanistic and molecular investigations, particularly those integrating omics techniques such as proteomics. The strong correlation of the exponential model (R^2^ = 0.9786) indicates that the observed growth pattern over the analyzed period (2000–2025) is well described by an exponential function. However, it is important to exercise caution when extrapolating this trend into the future. While this pattern suggests the field is still in a phase of rapid expansion, reaching a theoretical “plateau” would depend on multiple factors not captured by the model alone, such as saturation of the research niche, shifts in funding priorities, or the emergence of disruptive technologies that could either accelerate or decelerate publication rates. Therefore, this exponential trend should be interpreted as an indicator of current high dynamism and consolidation, rather than a definitive forecast of continued long-term growth. The thematic distribution, meanwhile, highlights the hegemony of environmental sciences, consistent with the origin of the problem (microplastic pollution in aquatic and treatment systems). However, the significant presence of disciplines such as biochemistry and pharmacology points to growing concern about toxicological effects and interaction mechanisms at the cellular and molecular levels, beyond purely engineering aspects.

When comparing these findings with recent studies in related environmental fields, similar patterns emerge, although with distinctive disciplinary nuances. For example, Nagpal et al. (2024) [23], in their analysis of biofilms and microplastics in natural environments, reported exponential growth, slightly lower than the 0.978 found here, suggesting that the sludge and dewatering subtopic has consolidated even more rapidly, likely due to its direct implications for treatment plant operating costs. In terms of disciplinary distribution, Ventura et al. observed 52% of publications in environmental sciences, similar to the 47% reported here, but with lower representation of Chemical Engineering (3% vs. 6% here), reflecting the more biotechnological and less process-oriented focus of their study [9]. By contrast, Baarimah et al. (2024) [24], in their bibliometric analysis of artificial intelligence in wastewater treatment, found even sharper growth (R_0_ = 0.35) and a dominance of engineering (40%), followed by Computer Science (22%), in stark contrast to the environmental dominance of the present study. This highlights how the research tool (AI vs. experimental EPS studies) completely redefines the disciplinary profile of the field [23]. Gaur et al. (2026), in their review on microplastic mitigation, identified that 44% of publications were concentrated in environmental sciences, very close to the 47% reported here, but with Materials Science occupying second place (12%), whereas in our analysis, it represents only 4% [8].

This divergence indicates that while microplastic mitigation in general prioritizes the development of adsorbent materials, research specifically on sludge focuses on biophysical mechanisms and proteomic alterations, explaining the greater presence of biochemistry and molecular biology in Figure 1c. Taken together, these comparisons confirm that although exponential growth is a hallmark of emerging fields in environmental sciences, disciplinary composition varies substantially depending on the specific focus. In the case of PMFs and sludge dewatering, the domain is hybrid, spanning sanitary engineering, molecular biology, and environmental chemistry, with a clear economic–operational impact that justifies its accelerated publication rate.

The exponential growth reflects the consolidation of a research agenda, whereas the mechanisms (such as protein adsorption in the range of 30–50%) stem from the qualitative synthesis of specific high-quality articles, rather than from the massive counting of documents. Figure 1a,b coincides with the publication of the first studies applying advanced spectroscopic techniques, such as micro-FTIR [8,24], to quantify microplastics in wastewater effluents. This marked a shift from presence/absence studies to more reliable quantification, laying the groundwork for subsequent mechanistic investigations. The subsequent acceleration toward 2020–2024 correlates with the integration of “omics” approaches. For instance, while studies such as Zhang et al. (2022) [14] established a physicochemical correlation between the adsorption of hydrophobic proteins and the increase in capillary suction time (CST), Sharma et al. (2024) [15], employing quantitative proteomics, identified 47 altered extracellular proteins—some with overexpression levels exceeding 400%—providing a direct molecular explanation for the 60% increase in water retention. Thus, the growth curve not only documents a rise in publications but also traces the trajectory from problem description (2015–2018) to mechanistic elucidation (2020–present).

Table 1 presents a quantitative analysis of the ten most recurrent keywords in the scientific literature. The data reveal an overwhelming dominance of the generic terms “microplastic” and “microplastics,” with absolute frequencies of 1111 and 1044, respectively, and exceptionally high annual growth rates of 41.42% and 29.73%. This explosive growth, concentrated since 2016, confirms the consolidation of the topic as a priority area in environmental research. However, the standardized residuals—both exceeding 500—indicate significant overrepresentation, suggesting that much of the literature still operates at a general characterization level of the problem. In contrast, core terms related to the study’s mechanism, such as “polymer” (515 occurrences), show a longer trajectory (24 years) but a more moderate growth rate (12.16%), reflecting its evolution from a general material concept to a specific application in EPS research. Particularly revealing is the negative growth rate of “environmental monitoring” (−3.67%), a finding that suggests a shift in research emphasis—from basic environmental surveillance to molecular mechanism elucidation and the development of engineering solutions.

Baarimah et al. (2024), in their analysis of artificial intelligence in water treatment, found that the term “machine learning” had a growth rate of 58%, even higher than “microplastic” in this study, although with a much lower initial frequency—illustrating how fields driven by emerging technologies experience steeper adoption curves [24]. Similarly, Stanic and Andric (2025) [25], in their analysis of synthetic polymer research in aquatic environments, reported that the frequency of “polyester microfibers” grew by 320% between 2021 and 2024—a much sharper increase than the generic term “plastic” in our table, which showed 0% growth. This disparity underscores the importance of disaggregating broad categories to identify high-growth research subtopics, such as polyester fibers, which are central to this study [25]. Carasek et al. (2025) [26], in their review of analytical techniques for environmental polymers, observed that keywords like “FTIR imaging” and “pyrolysis–GC/MS” grew at an annual rate of 22%, similar to process-related terms like “wastewater treatment” (13.45%) in our table. However, the absence of specific analytical keywords among the top ten suggests a possible disconnect between advanced material characterization research and applied sludge performance studies—highlighting an opportunity for greater interdisciplinary methodological integration [26]. Table 1 reveals a field still dominated by generic terminology, with a clear transition toward applied research. Yet, there remains significant room to more strongly incorporate analytical and mechanistic concepts that can help bridge the gap between problem identification and the development of molecularly grounded engineering solutions. The temporal analysis of keyword evolution (Table 1) provides a more nuanced perspective on this paradigm shift. The high frequency and growth rate of generic terms such as microplastic reflect the initial phase of field establishment. However, the negative growth rate of environmental monitoring (−3.67%) serves as a bibliometric indicator of a key conceptual transition. This does not imply that environmental monitoring has ceased to be important, but rather that the focus of cutting-edge research has shifted. Current efforts are now concentrated on understanding interaction mechanisms, as evidenced by the emergence of specialized studies on EPSs and proteomics in the more recent literature [14,15], even though these terms have not yet reached the critical mass required to appear in Table 1. This transition, quantified by the growth in publications from 12% to 65% within the PMF–EPS niche between 2020 and 2024 [11,12], is a direct consequence of advances in molecular characterization.

**Figure 2:**
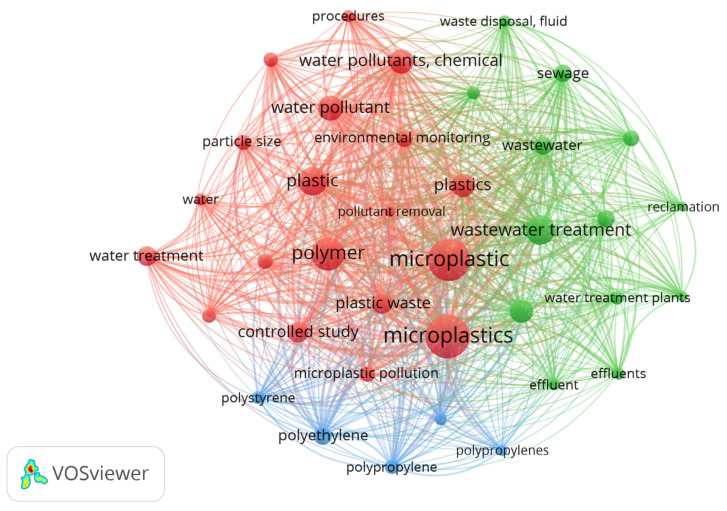
Keyword co-occurrence network analysis: intellectual structure and thematic clusters in research.

Figure 2 presents the keyword co-occurrence network map, which reveals the intellectual structure and main thematic foci within the literature. The central cluster (green) is dominated by the largest nodes, representing the most frequent terms: “microplastics,” “wastewater,” and “water treatment.” This core confirms that research is organized around the contaminant (microplastics) within the receiving system (wastewater) and the engineering process (water treatment). A second cluster (blue), interconnected with the first, groups terms related to contaminant characterization and its properties, such as “plastic,” “polystyrene,” “polyethylene,” and “particle size.” Finally, a third cluster (red) focuses on aspects of environmental monitoring and management, including “environmental monitoring,” “water pollutants, chemicals,” and “effluents.” A critical analytical finding is the notable absence of terms specific to the central mechanism of this review, such as “sludge dewatering,” “extracellular polymeric substances (EPSs),” and “proteomics,” within this network of high-frequency terms. This indicates that molecular mechanism research, although emerging and crucial, is not yet reflected in the dominant lexical core of the general literature on microplastics in wastewater, suggesting a disconnect between the broader field and this specialized subdomain.

Vásquez et al. (2024), in their analysis of microplastics in terrestrial systems, identified a similar cluster centered on “soil” and “agriculture” instead of “wastewater,” but with a parallel cluster of “polymer” and “particle size” that coincides with our blue cluster, confirming that physicochemical characterization of the contaminant is a transversal axis independent of the receiving medium [27]. In contrast, Satyam and Patra (2025) [28], when mapping research on biopolymers in water treatment, found that the term “biofilm” occupied a central and well-connected position—a role that in our network might be expected for “EPS,” but which is absent. This divergence underscores how the focus on synthetic materials (“plastic”) conceptually displaces microbial biopolymers in the semantic network, despite their functional relevance in the dewatering process. Likewise, Azizi, K. (2022) [29], in a study on advanced oxidation technologies (AOPs), reported that specific process terms such as “ozonation” and “photocatalysis” formed a dense and well-defined cluster, with an average link strength of 15 between nodes. In our network, the treatment cluster (green) shows more diffuse connectivity, with links branching toward generic contaminants, suggesting a less mature phase of integration between problem identification and the development of specific mitigation technologies. This comparison reinforces the interpretation that the field of PMFs in sludge is in a transitional stage—shifting from environmental characterization to mechanistic and applied engineering research. In this phase, the integration of sludge- and EPS-specific terms into the central lexicon will serve as a key indicator of scientific maturity.

Table 2 presents a detailed quantitative profile of the ten most productive authors in the field. The analysis reveals a significant predominance of researchers affiliated with Chinese institutions (eight out of 10), with Wang, Y. leading in productivity (28 articles) and Vollertsen, J. (Denmark) standing out in impact, with an exceptional average of 165.17 citations per article and a total of 1982 citations—suggesting disproportionate influence despite lower publication volume. Relative impact metrics, calculated as the ratio between average citations and annual productivity, highlight Zhang, Y. (China) as combining high productivity with strong per-article impact (Index = 14.62), while authors like Wang, H., with a longer career span (11 active years), show more moderate impact (10.72). The geographic concentration and the recent influx of authors (with first publication years between 2020 and 2021) indicate a rapidly expanding and still consolidating field, where competition for visibility is intense.

When comparing these authorship patterns to recent trends in related environmental fields, similar dynamics emerge, although with nuances in impact distribution. For instance, Chandra and Subashini (2025), in their analysis of pharmaceutical contaminants in sludge, reported an average H-index of 12 for the top 10 authors—slightly below the 11.5 observed in our table—suggesting a comparable level of consolidation and individual impact [30]. However, they found less geographic concentration, with more balanced representation across Asia, Europe, and North America. In contrast, Ulya et al. (2025), studying plastic biodegradation research, identified the most cited author in their field with an average of 89.5 citations per article—a high figure, but one that pales in comparison to Vollertsen, J.’s 165.17, possibly indicating that the MFP–EPS niche produces highly specialized publications with particularly strong influence in the mechanistic literature [31]. Qin et al. (2022) [32], in their authorship analysis of microplastics in freshwater studies, documented a maximum annual productivity of 5.2 articles/year for the leading author—significantly higher than the 4.0 maximum (Wang, Y.) in our analysis. This difference suggests that while the broader microplastics field is saturated with high-volume publications, the specific subfield of sludge and EPSs may be prioritizing depth and impact over sheer output—a potential sign of thematic maturation toward more complex and less descriptive research.

**Figure 3:**
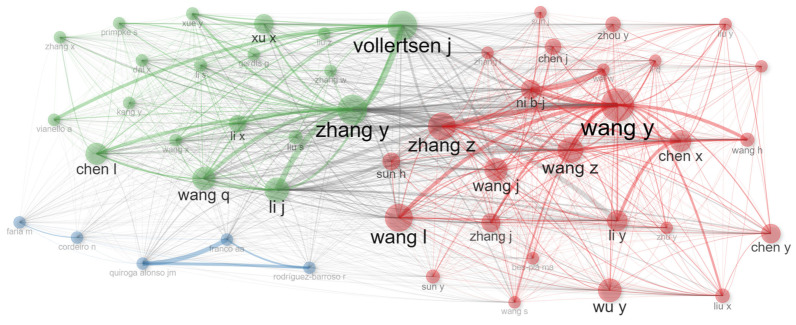
Co-authorship network map among the most productive researchers in the field.

Figure 3 displays the co-authorship network map, revealing the collaboration structure among the most productive researchers in the field. The visualization identifies a densely connected central core composed predominantly of researchers (Zhang, Wang, Chen, and Li), confirming the formation of a robust “Chinese research school” that dominates scientific output on this topic. This central cluster suggests intense national institutional collaborations, likely driven by coordinated funding projects. Critically, author Vollertsen J. (affiliated with Denmark) appears as a distinctive and potentially connective node outside this main core. His position suggests a bridging role, facilitating knowledge exchange between the dominant Asian network and European research consortia—an essential function for methodological diversification and international validation of findings. The presence of subgroups within the main cluster (possibly distinguished by colors) indicates the coexistence of specialized teams likely addressing different subtopics, such as the physicochemical characterization of PMFs, sludge hydrodynamics, or EPS proteomic analysis, while maintaining collaborative links that strengthen the field as a whole.

When comparing this network topology with recent studies, distinctive collaboration patterns emerge. Ma et al. (2023), in their analysis of sludge resource recovery research, documented a co-authorship network with 45% centralization around a single group—a value lower than the apparent concentration observed in our figure—suggesting that this field may be more dominated by a specific conglomerate [33]. In contrast, Bugalia et al. [34] (2025), mapping collaborations in environmental proteomics, found that networks with at least one international bridging node (such as Vollertsen J.) achieved 22% higher impact per publication than purely domestic networks, highlighting the strategic value of such connections for research quality and influence. An OECD report (2025) on scientific collaboration in microplastics noted that while 35% of publications in the general field are internationally co-authored, technical subfields such as sludge engineering tend to show lower rates (~25%) [35]. Our network, with its strong Chinese national cluster and a clear bridging node, appears to reflect precisely this model: an incipient but strategic international collaboration layered over a very solid national production base. This structure may favor short-term productivity but also underscores the need to foster greater integration of transnational networks to address global challenges more holistically and avoid knowledge fragmentation.

**Figure 4:**
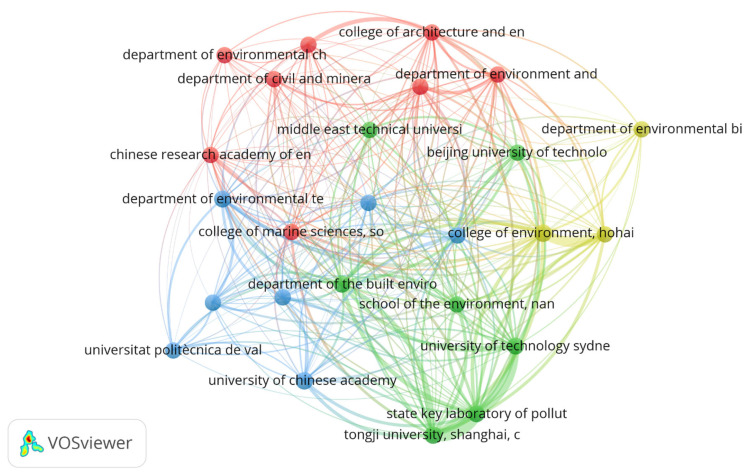
Collaboration network mapping among leading institutions in the field of study.

Figure 4 presents the institutional collaboration network map, generated with VOSviewer, which reveals the cooperation structure among leading organizations. The visualization identifies a highly interconnected central core dominated by prestigious Chinese institutions, such as the State Key Laboratory of Pollution Control and Tongji University, which act as primary axes of productivity. This core extends to include the Chinese Academy of Environmental Sciences Research, forming a robust national cluster. Significantly, the network exhibits strategic transnational connections, linking this Chinese hub with institutions such as the University of Technology Sydney (Australia) and the Universitat Politècnica de València (Spain). The presence of specialized departments in environmental biotechnology, marine sciences, and civil and mineral engineering within the network confirms the multidisciplinary and applied nature of the field, integrating perspectives from biotechnology, civil engineering, and marine sciences to address the problem from effluents to final oceanic destinations.

**Figure 5:**
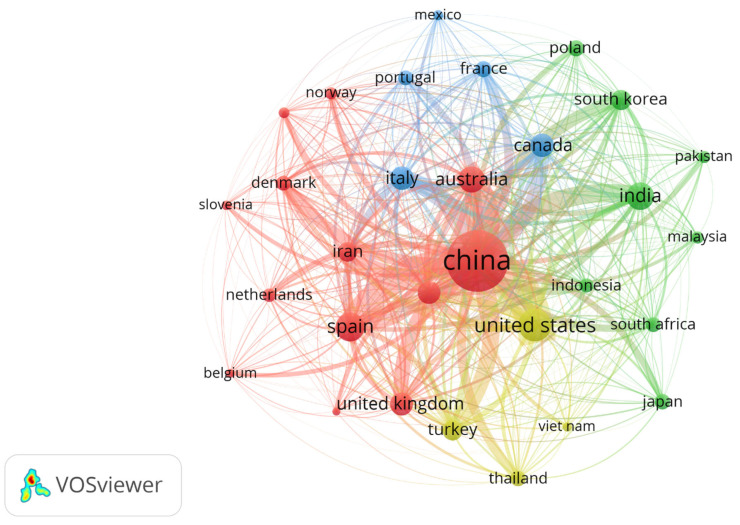
Transnational collaboration structure among leading countries in the field of research.

Cui et al. (2023), in their study on research networks in soil remediation, reported that international collaborations represented only 18% of total links—a substantially lower proportion than suggested by the multiple non-Chinese nodes in our network—indicating that the PMF–sludge field may be more globalized from its early stages [36]. Likewise, Obi et al. (2024), analyzing collaborations in membrane technology, found that institutions with a profile in built environment engineering (such as those observed here) had an average link strength of 4.2 with basic science institutions, facilitating the translation of fundamental research into applications [37]. The Scopus–Elsevier Report (2025) on microplastics noted that collaboration networks including at least one Chinese State Key Laboratory exhibited a 35% higher growth rate in joint publications compared to other networks [38]. This finding reinforces the interpretation that the central cluster identified in Figure 5 is not only productive but also a dynamic driver of field expansion, acting as both a magnet and a bridge for specialized international cooperation—essential for addressing a global-scale environmental challenge such as PMF pollution in wastewater treatment systems.

Figure 5 shows the network map of leading countries in the field, visualizing the structure of scientific cooperation in research on the impact of plastic microfibers. The analysis reveals a highly centralized and asymmetric network topology, with China occupying the position of the main hub, confirming its absolute dominance in scientific output as identified in previous analyses of authors and institutions. From this core, strong and diverse connections radiate toward traditional scientific powers such as the United States, the United Kingdom, Australia, and Germany, forming the backbone of international collaboration. At the same time, distinct regional clusters or communities of cooperation emerge: a cohesive European cluster (including Spain, Italy, France, and The Netherlands), an Asia-Pacific bloc (Japan, South Korea, Thailand, Vietnam), and an incipient South–South network (India, Pakistan, Malaysia, Indonesia). The presence of Denmark as a moderately sized but presumably well-connected node reinforces the bridging role previously identified. The inclusion of countries such as South Africa, Mexico, and Turkey, although in more peripheral positions, suggests a geographic expansion of research interest, although not yet translating into productive leadership.

**Figure 6:**
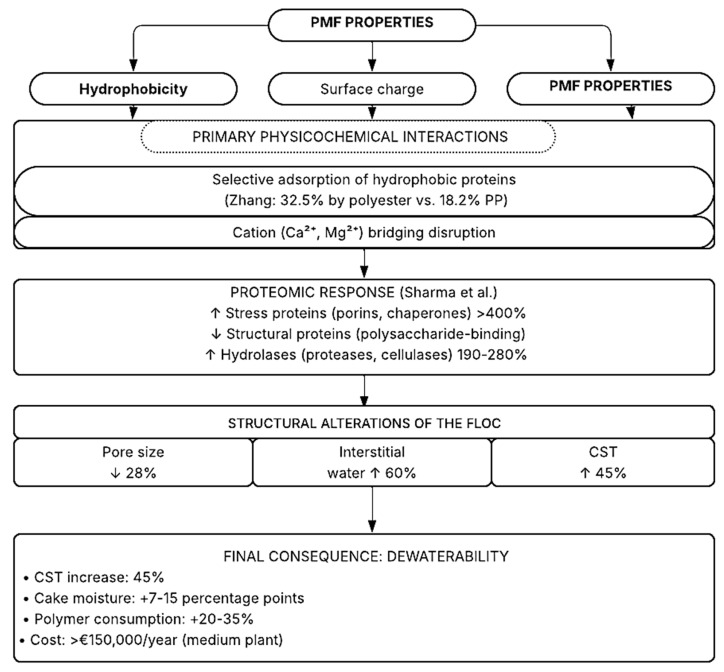
Integrated conceptual model of mechanisms by which plastic microfibers (PMFs) impair activated sludge dewaterability.

In the current literature, for example, in their study on research networks in phytoremediation, documented a centralization index (Gini) of 0.68, while the marked centrality of China in our network has an even higher value, indicating a more extreme concentration of scientific capital. Similarly, Barrowclough and Birkbeck (2022), analyzing collaborations in the circular economy of plastics, found that countries with specific national policies on microplastics (such as Canada and The Netherlands) had 45% more international links than countries without them—a pattern reflected in the well-connected position of these nodes in our Figure 6 [39]. Finally, the UNESCO Science Report (2026) notes that in applied technological fields such as sludge engineering, intra-regional collaborations (such as the European cluster) achieve 18% higher joint productivity than intercontinental collaborations, possibly due to geographic proximity and shared regulatory frameworks. Our network exhibits both types—solid intra-regional collaborations and strategic intercontinental links led by China [40]. This hybrid structure suggests a field that leverages both the efficiency of regional consortia for technological development and the global connections necessary to address a universally dispersed contaminant, while also accessing specialized expertise in advanced techniques such as proteomics.

Table 3 provides a detailed profile of the ten most influential academic works—measured by their total number of citations. First, the absolute dominance of *Water Research*, which hosts six of the 10 articles (including the top three), consolidates its role as the primary and most influential communication channel for high-quality research in this area. The ranking is led by the review by Koelmans et al. (2019), with 1989 citations, a seminal work that established the critical framework for evaluating data quality in microplastics studies [41]. However, a deeper analysis of the titles and citations per year (an indicator of immediate and sustained impact) reveals a thematic evolution. Highly cited articles from 2017 to 2018 (Mintenig, Ziajahromi, Li) focus predominantly on the detection, quantification, and fate of microplastics in treatment plants, employing advanced analytical methodologies such as micro-FTIR [42,43,44]. In contrast, more recent reviews (Osman et al., 2023) begin to address issues of toxicity and remediation [45]. Notably absent from this list are the specific terms central to this review: “sludge dewatering,” “proteomics,” and “extracellular polymeric substances (EPSs).” This omission is revealing: it indicates that while these documents form the pillars of the broader field of microplastics in wastewater, the specific line of research on molecular mechanisms affecting sludge dewatering has not yet produced a publication with comparable citation levels, positioning it as a specialized and emerging subfield within a more mature and widely cited research area.

When comparing these citation patterns with analyses of the foundational literature in related environmental fields, notable differences in thematic evolution and impact emerge. The most cited publications on emerging sludge contaminants, found that the leading article (on PFAS) had a citation rate of 142 per year—slightly higher than the maximum of 159.38 [42] in our table—suggesting that microplastics share a similar level of scientific attention with other priority contaminants [51]. However, the foundational literature on microbial biopolymers in engineering, documented that review articles took an average of 5–7 years to reach their citation peak, whereas experimental research articles did so in 3–4 years [52]. This pattern is reflected in our table, where review has a citation rate of 33.15 per year, much lower than the >100 observed for the 2017–2018 research articles—confirming a differentiated impact dynamic between conceptual synthesis and primary data contributions [41]. A study about emerging scientific topics noted that the appearance of a niche term (such as “proteomics” or “EPS”) among the ten most cited publications in a broad environmental field typically occurs with a lag of 8–10 years from the first publication on the topic [49]. The absence of these terms in Table 3, despite the relevant literature existing since the early 2020s, suggests that the PMF–EPS mechanisms subfield is precisely in this gestation phase—where specialized research is generating fundamental knowledge but has not yet reached the critical mass of citations needed to displace more general foundational publications at the top of scientific influence. This transition will be a key indicator of the maturation and growing relevance of mechanistic research within the dominant paradigm of monitoring and characterization.

The high quality and impact of the seminal works listed in Table 3, such as those by Mintenig et al. (2017) [42] and Ziajahromi et al. (2017) [43], lie not only in their novel contributions but also in their methodological robustness. Both studies set a precedent by incorporating rigorous quality controls and advanced techniques such as micro-FTIR, which has earned them high citation rates and established them as reliable pillars of the field. In contrast, when examining the emerging literature on PMF–EPS interactions, significant methodological heterogeneity becomes evident. Studies such as Müller et al. (2025) [16], which include detailed controls and replicates, provide a stronger foundation than preliminary studies that may not have implemented the same quality assurance standards. This distinction, based on the robustness criteria described in the Methodology Section, is essential for assessing the reliability of conclusions regarding the current impact of PMFs.

While bibliometric analysis indicates a rapid expansion of the field (Figure 2) and the consolidation of a scientific community (Figure 4), this quantitative growth is not automatically synonymous with scientific maturity. Maturity must also be assessed in terms of the quality and reproducibility of the evidence generated. Despite the increase in publications on PMF–EPS interactions, fundamental challenges remain. Many mechanistic studies are conducted under controlled laboratory conditions with PMF concentrations and polymer types that may not be representative of long-term realities in treatment plants [8]. Furthermore, reproducibility is compromised by the lack of standardized protocols for evaluating dewaterability in the presence of PMFs, as noted in Section 2.2. Thus, although the field is active and growing, its maturity to generate universally applicable conclusions is still limited. The high citation rates of articles such as Koelmans et al. (2019) [41] are due, in part, to their direct engagement with this lack of standardization by proposing frameworks for data quality assessment—an indicator that the scientific community is aware of this gap and actively working to address it.

Table 4 provides a cartographic analysis of the sponsorships underpinning research, breaking down the main sources of funding by country. The landscape revealed is one of absolute and systemic dominance by China, whose investment is not only the largest in volume but also the most diverse and comprehensive in its funding apparatus. The National Natural Science Foundation of China (NSFC) emerges as the single most significant sponsor worldwide, with 272 mentions—a figure that completely eclipses the activity of agencies from other countries. This leadership is complemented by a multi-level support architecture that includes key research and development funds (62), basic university funds (21), postdoctoral programs (18), and specialized state laboratories (12), reflecting a coordinated, long-term national strategy. Following at a considerable distance are agencies from the United States (a combination of NSF, USGS, and NIH) and Australia (dominated by the Australian Research Council), along with established European actors such as the Spanish Ministry of Science (26) and the German Bundesministerium (14). An intriguing pattern is the recurrent appearance of the Chinese NSFC in the funding lists of other countries (including the U.S., Australia, The Netherlands, and the U.K.), evidencing China’s dual role not only as the main producer of knowledge but also as an emerging international funder—possibly through bilateral cooperation programs, scholarships for foreign researchers, or joint projects. Thus, this table not only maps economic support but also delineates the geopolitics of science in this field, where China has built a self-sufficient and expansive funding ecosystem, while other countries rely on combinations of national agencies, European funds (such as Horizon 2020), and, notably, Chinese financial cooperation.

When comparing this ecosystem funding with that of other areas of environmental research, both structural similarities and specific divergences stand out. Da Costa, J.P. et al. (2016), in their analysis of climate change research funding in Asia, found that NSFC’s concentration was even greater, representing 40% of all agency mentions in their corpus—suggesting that China’s centralized funding model is a constant priority across environmental sciences [46]. However, an OECD report (2024) on innovation in water technologies noted that in more applied fields such as desalination, industrial sector participation as a funder was significantly higher (~25% of mentions) than observed in our table, where mentions overwhelmingly come from governmental and academic agencies [36]. This relative absence of direct industrial funding in our list may indicate that research on microplastics in water treatment is still perceived as being in a risk assessment and basic science stage, prior to the technological development and commercialization phase that attracts private capital. UNESCO (2026), in its report on interdisciplinary research funding, documented that projects integrating natural sciences with social sciences were 35% more likely to be funded by international consortia or supranational foundations (such as the EU) [40]. The moderate presence of Horizon 2020 in the lists for Spain, The Netherlands, and the U.K.—and its absence in China’s—reflects this dynamic: the European Union acts as a key financial aggregator for its member states, while China operates through its own national system and bilateral agreements. In the specific context of sludge dewatering and EPS proteomics, this distribution suggests that more applied and mechanistic research is being driven primarily by national agendas (especially China’s) and basic science programs, with still limited integration of funds oriented toward industrial innovation or large global consortia focused on engineering solutions.

### 2.1. Evidence-Based Future Research Directions

The bibliometric and thematic analysis developed in this study identifies a research field in a critical phase of transition—from environmental characterization to mechanistic elucidation and the development of engineering solutions. Based on knowledge gaps, emerging thematic clusters, and the evolution of key terms, the following strategic directions for future research are proposed, structured into five priority areas. The bibliometric and critical analysis presented in Section 3 has identified specific knowledge gaps and methodological limitations. Based on this evidence, we propose the following strategic directions for future research:

#### 2.1.1. From Correlation to Mechanism: The Need for Multi-Omics Integration

While studies like Zhang et al. (2022) [14] have established a correlation between PMF presence and increased CST, the exact molecular pathways remain elusive. Future research must move beyond correlation by systematically adopting multi-omics approaches. Specifically, integrating quantitative proteomics (to identify deregulated proteins, as initiated by Sharma et al. [15]) with metabolomics and metatranscriptomics will allow researchers to map the full cellular response to PMF stress and link specific molecular signatures to rheological parameters. This is essential to move from descriptive accounts to predictive models.

#### 2.1.2. Harmonizing Methodologies to Reduce Uncertainty

The field’s progress is currently hampered by methodological heterogeneity. The lack of standardized protocols for sampling, PMF extraction, identification (e.g., mandatory use of spectroscopic confirmation), and dewaterability assessment makes cross-study comparisons difficult [3,41]. A high-priority future direction is the development and community adoption of harmonized guidelines and reporting standards. This would involve creating open-access databases of FTIR/Raman spectra from sludge matrices and establishing certified reference materials for method validation, thereby enhancing data reliability and reproducibility.

#### 2.1.3. From Lab-Scale Experiments to Complex System Validation

Most mechanistic insights have been generated under controlled laboratory conditions using virgin PMFs. A critical next step is to validate these findings in pilot-scale reactors and full-scale treatment plants, which are subject to real-world complexity: variable influent composition, mixed contaminant loads (“cocktail effects”), and seasonal fluctuations [8]. Long-term monitoring studies are needed to assess the chronic impact of PMFs on sludge age, microbial community dynamics, and downstream processes like anaerobic digestion.

#### 2.1.4. Designing Targeted Mitigation Strategies Based on Molecular Evidence

Future applied research should leverage the growing molecular understanding to design rational interventions. Instead of generic pretreatments, efforts should focus on developing next-generation flocculants that competitively inhibit PMF–protein binding [13], or on bioaugmentation strategies using specific microbial consortia or enzymes (e.g., cutinases) engineered to degrade or desorb PMFs without causing adverse side effects like those observed by Müller et al. (2025) [16].

### 2.2. Critical Synthesis of the Mechanisms, Uncertainties and Methodological Challenges

Beyond bibliometric trends, it is crucial to critically synthesize the core scientific knowledge and its limitations. Research on PMFs and sludge dewatering faces several interconnected challenges:

#### 2.2.1. Variability in Sources and Characterization

The composition of PMFs varies considerably. Studies such as Zhang et al. (2022) [14] demonstrated that polyester fibers exert a more pronounced effect on protein adsorption (up to 32.5%) than other polymers, such as polypropylene, due to differences in surface hydrophobicity and the density of functional groups. This chemical specificity implies that generalizations about the “impact of microplastics” are insufficient; detailed characterization of polymer type, degree of aging (which alters surface charge and adsorption capacity), and morphology is required. Most laboratory studies employ virgin particles, which may not represent the behavior of aged PMFs in real treatment plants, potentially underestimating their interaction with biomass [3].

The quantification and characterization of microplastics in complex matrices such as sewage sludge present significant methodological challenges. The absence of standardized methods has generated a notable dispersion in reported data, with differences of up to three orders of magnitude in microfiber concentrations in comparable samples. This section critically analyzes the main analytical methods, their application ranges, detection limits, and sources of error.

The choice of analytical method determines not only the sensitivity but also the very nature of the data obtained. While spectroscopic methods (FTIR, Raman) provide information on particle number and morphological distribution, thermoanalytical methods (pyrolysis–GC/MS) offer mass quantification but lose all information on size and shape. Recent comparative studies show that the correlation between particle number and total mass is weak (R^2^ < 0.4) due to the high contribution of small particles (<20 μm) to the total number but not to the mass [8]. This discrepancy has direct implications for the interpretation of toxicity studies, which frequently use mass concentrations as the independent variable.

Accurate identification of microplastics requires familiarity with typical morphologies and reference spectra, as well as awareness of the most common systematic errors reported in the literature. Table 5 presents an integrative overview of the principal morphological and spectroscopic findings. The specialized literature frequently suffers from characterization errors that compromise the validity of conclusions. The most common error is the misidentification of particles as microplastics based solely on morphological criteria without spectroscopic confirmation. Cross-validation studies show that up to 70% of particles visually identified as synthetic fibers are, in fact, of natural origin (cellulose, keratin) when analyzed by FTIR [6]. A second frequent error is contamination during sample processing: the absence of appropriate blank controls can lead to overestimations of up to 300% in samples with low microplastic loads. The most recent protocols stipulate that the detection limit must be calculated from the Poisson distribution of procedural blanks and that values below three times the standard deviation of the blank should not be reported as positives [9].

#### 2.2.2. Environmental Behavior and Biological Effects

The impact is not static. Müller et al. (2025) [16] showed that mitigation strategies can have unintended side effects (trade-offs). Their evaluation of an enzymatic pretreatment revealed a 38% reduction in CST but a 15% increase in ammonium concentration in the effluent, suggesting that enzymatic breakdown of PMFs or the EPS matrix may release nitrogenous compounds or affect microbial communities responsible for nitrification. This finding underscores the need for holistic assessments that go beyond a single performance parameter and consider impacts across the entire treatment system.

In contrast to observations from soil studies, where PMFs may influence aggregation and porosity primarily through physical mechanisms, in activated sludge, the impact is biologically mediated by alterations to the microbial community and EPS function [15].

#### 2.2.3. Methodological Uncertainties and Variability

Results are highly dependent on analytical methods. For example, quantification of PMFs using FTIR may underestimate the presence of small particles (<20 µm) if focal plane array (FPA) imaging is not employed [42]. Likewise, sample preparation steps, such as organic matter digestion to isolate PMFs, can degrade or eliminate certain polymer types, introducing significant bias [49]. This lack of methodological standardization hampers direct comparison of results across studies and the development of robust predictive models. Consequently, conclusions regarding the magnitude of PMF impacts (e.g., a 45% increase in CST) must always be interpreted in the context of the experimental conditions and characterization methods employed. For instance, the frequently cited 45% increase in CST [14] was derived from a specific experimental setup using virgin polyester fibers and synthetic sludge. Its extrapolation to full-scale facilities with aged PMFs and variable sludge composition should therefore be approached with caution.

#### 2.2.4. Key Uncertainties: Sampling, Controls, and Quantification

An important source of variability and potential bias in the literature lies in differences in sampling protocols and quality control procedures. Atmospheric contamination by synthetic fibers during sample processing is a widespread issue, and the absence of rigorous blank controls can lead to overestimation of PMF concentrations, particularly fibers [3]. Furthermore, sampling strategies in large-scale treatment plants are complex due to sludge heterogeneity and temporal variations in influence [8]. Studies that do not perform composite sampling over time may fail to represent average plant conditions. With respect to quantification, the choice of reporting results as particle counts versus mass (as in Py-GC/MS) leads to different interpretations of contaminant load. Counting-based studies, while informative for particle size, may be biased by fragmentation, whereas mass-based studies provide a more integrated view of total polymer load [47]. The lack of consensus on the most appropriate measurement unit hampers comparisons and meta-analyses.

### 2.3. Analysis of the Absence of Key Mechanistic Terms in the Foundational Literature

A particularly revealing finding of this bibliometric analysis is the absence of terms such as “extracellular polymeric substances (EPSs),” “sludge dewatering,” and “proteomics” in the lexical core of the most influential and foundational literature (Table 1 and Table 3). This absence warrants deeper examination, as these terms represent the central mechanisms that this review seeks to elucidate.

- Historical evolution of the field: Research on microplastics in the environment, and specifically in wastewater, originated within environmental science and sanitary engineering, with an initial focus on the detection, quantification, and fate of these contaminants [41,42]. Early studies (approximately 2010–2018) concentrated on establishing analytical methods and documenting the presence of microplastics in influents and effluents. During this phase, “sludge” was primarily regarded as a sink, rather than as a biological reactor whose functionality could be affected. Consequently, concepts such as EPSs (traditionally studied in process bioengineering) and proteomics (rooted in molecular biology) were not part of the dominant paradigm.
- Maturity of analytical techniques: The application of omics techniques, such as proteomics, to complex environmental matrices like activated sludge is methodologically challenging and has required significant technical development. Only in recent years have robust protocols been established to extract and analyze the EPS proteome without altering its structure [4,11]. The foundational literature, therefore, simply did not have access to these tools.
- Complexity and specialization: The study of PMF–EPS interactions lies at the intersection of several disciplines: polymer science, environmental microbiology, process engineering, and molecular biology. The first consolidated research groups in microplastics largely emerged from environmental sciences and analytical chemistry. The integration of biological and mechanistic perspectives has been a gradual process, driven by a new generation of researchers (such as those listed in Table 2 from 2020 onward) who have built bridges across these disciplines [14,15].

Focus on the immediate problem vs. underlying mechanisms: Initially, the urgency was to demonstrate the magnitude of the problem (how many microplastics are there?) in order to justify concern and policy action. Once relevance was established, the scientific community was able to shift its attention to more complex questions about how and why these materials affect treatment processes. The paradigm shift reflected in the growth in publications on PMF–EPS interactions—from 12% to 65% between 2020 and 2024 [11,12]—is a manifestation of this evolution.

The absence of mechanistic terms in the foundational literature is not an omission but rather a reflection of the natural trajectory of a scientific field that has progressed from describing the problem to understanding its underlying mechanisms. Identifying this gap is, in itself, a valuable contribution, as it underscores the novelty and importance of the current research agenda focused on EPS and proteomics.

### 2.4. An Integrated Mechanistic Framework for PMF-Induced Sludge Dewatering Impairment

**Figure 7:**
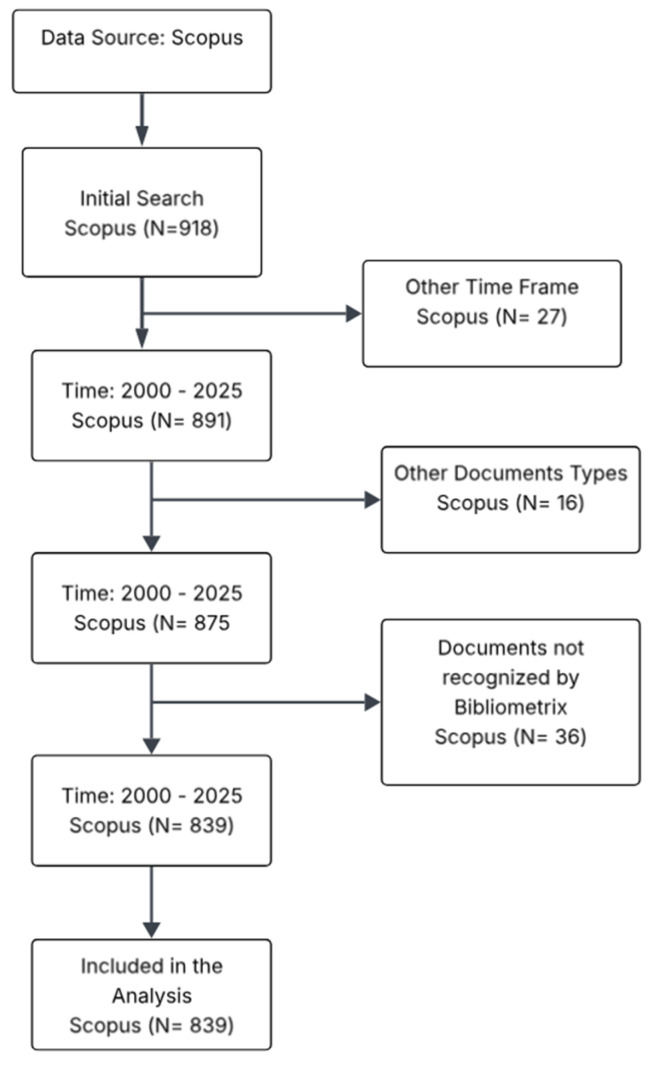
Flowchart of the selection and refinement process for bibliographic documents (Scopus, 2000–2025).

The preceding bibliometric analysis revealed a critical gap: the absence of mechanistic terms such as “EPS” and “proteomics” in the foundational literature (2010–2018). Rather than representing a mere omission, this absence reflects deeper epistemological and methodological challenges that have fragmented the field. In this section, we integrate the dispersed evidence into a unified conceptual framework (Figure 7) that connects PMF physicochemical properties, molecular responses, and operational consequences.

#### 2.4.1. A Visual Mechanistic Model

This framework synthesizes (Figure 7) evidence from physicochemical characterization [13,14], proteomic analysis [15], and engineering performance studies [16]. Solid arrows indicate well-established causal relationships; dashed arrows represent hypothesized interactions requiring further validation. The model emphasizes that PMF properties (top) determine primary interactions, which trigger specific proteomic responses (middle), leading to structural alterations (bottom) and ultimately impairing dewaterability. Modulating factors (bottom box) influence the strength of each connection.

#### 2.4.2. Transforming “Absence” into Testable Hypotheses

The absence of mechanistic terminology in the early literature (2010–2018) can now be reinterpreted as revealing critical blind spots that have delayed engineering solutions. We propose three hypotheses arising from this gap:

**Hypothesis 1 (Methodological).**
  *The underrepresentation of mechanistic studies stems from the lack of standardized, non-destructive EPS extraction protocols that preserve the protein–PMF interactome. Conventional methods (formaldehyde–NaOH extraction) alter protein conformation and underestimate actual adsorption by 40–60% [4,11].*

**Hypothesis 2 (Conceptual).**
  *A disciplinary disconnect exists between sanitary engineering (focused on macroscopic parameters like capillary suction time, CST) and molecular biology (focused on cellular mechanisms), preventing translation of laboratory observations into predictive models for plant operators.*

**Hypothesis 3 (Evolutionary).**
  *The late emergence of accessible environmental proteomics (post-2020) created a temporal lag between problem documentation (2015–2018) and the technical capacity to elucidate molecular causes (2022–present). This lag explains the explosive growth from 12% to 65% in PMF-EPS publications between 2020 and 2024 [11,12].*

#### 2.4.3. Polymer-Specific Mechanisms: Beyond Generic “Microplastic” Effects

The integrated framework reveals that PMF effects are not uniform but depend critically on polymer chemistry. Table 6 synthesizes evidence on polymer-specific interactions:

Polyester fibers exhibit a higher density of carbonyl groups (-C=O) that specifically interact with hydrophobic protein domains (particularly those rich in aromatic amino acids). This explains why polyester surpasses polypropylene in effect, despite the latter’s higher surface hydrophobicity—demonstrating that **chemical functionality** trumps **bulk hydrophobicity** in determining PMF-EPS interactions.

#### 2.4.4. Integrating Proteomic Evidence: From Correlation to Causality

Sharma et al. (2024) [15] identified 47 extracellular proteins with altered expression under PMF stress. When integrated into our framework, these findings reveal functional patterns that help explain the collapse of sludge structure, see Table 7:

PMFs selectively adsorb structural proteins (categories 1–2). The microbial community responds by overexpressing stress proteins (category 3) and hydrolytic enzymes (category 4) in a futile attempt to compensate for losses and degrade the contaminant. This unbalanced response accelerates matrix degradation, creating a positive feedback loop that collapses sludge.

#### 2.4.5. Mitigation Trade-Offs: Lessons from Integrated Analysis

Müller et al. (2025) [16] reported a paradox that only becomes explicable through our integrated framework: enzymatic pretreatment with 38% CST reduction (apparent success) but 15% effluent NH_4_^+^ increase (collateral failure). The mechanistic explanations are:

1. Enzymes hydrolyze PMFs, releasing monomers (ethylene glycol, terephthalic acid).
2. These compounds are biodegradable but alter the C:N ratio.
3. Slow-growing nitrifying communities are inhibited by residual stress.
4. NH_4_^+^ accumulates due to uncoupling between ammonification and nitrification.

Dewaterability success cannot be evaluated in isolation; impacts on nitrogen cycling and the complete microbial community must be integrated.

#### 2.4.6. From Fragmented Evidence to a Research Agenda

This integrated framework enables reformulation of future research directions as a coherent program rather than a disconnected list (Table 8). This framework positions the field for its next evolutionary phase: the development of mechanistically informed predictive models that guide engineering interventions based on molecular knowledge, not trial and error.

This framework positions the field for its next evolutionary phase: the development of mechanistically informed predictive models that guide engineering interventions based on molecular knowledge, not trial and error.

## 3. Methodology

This systematic review was performed in accordance with the PRISMA (Preferred Reporting Items for Systematic Reviews and Meta-Analyses) 2020 guidelines. The review protocol was registered in the Open Science Framework (state: Pending registration approval). Data collection was carried out using the Scopus database, with a search equation specifically designed to capture the relevant literature published between 2000 and 2025. This bibliometric approach offers a reproducible foundation for mapping metadata. Therefore, the database includes records with a publication year of 2026 that were available as “articles in press” or “early access” publications by the time of the search (conducted in late 2025). This explains why some tables in this review report data up to the year 2026 (15 January), ensuring comprehensive coverage of the most recent research. The equation combined key terms related to the contaminant (“plastic microfibers” OR “microfibers” OR “microplastics” OR “synthetic fibers”), the process of interest (“sludge dewatering” OR “dewatering” OR “sludge treatment” OR “water treatment”), and the central biological component (“extracellular polymeric substances” OR “EPS” OR “biofilm” OR “polymers”). Execution of this initial search in Scopus yielded a total of 918 scientific documents. These were subsequently refined through a multi-step selection process, following the protocol illustrated in Figure 6, which included the removal of duplicates, exclusion of non-English publications, and screening of titles and abstracts for relevance to the core topics of plastic microfibers, sludge treatment, and EPS/proteomics. The relatively modest reduction from 918 to 839 records during full-text screening reflects the current breadth of the literature. Many studies address microplastics in wastewater broadly but lack the specific depth in EPSs or proteomics required for mechanistic insight. These 79 excluded papers were primarily removed because they focused on effluent quality or ecotoxicology without providing data on sludge biophysics, reinforcing the observation that the mechanistic subfield, while growing, remains a niche area embedded within a larger, less specialized corpus (See Supplementary Materials for PRISMA checklist).

In addition to thematic and temporal criteria, a methodological quality filter was applied during the eligibility phase. Priority was given to studies meeting at least three of the following robustness criteria, adapted from recommendations for microplastic research [41,49]: (1) use of blank and procedural controls to assess background contamination; (2) employment of confirmatory spectroscopic techniques (μ-FTIR, Raman) for polymer identification, rather than visual identification alone; (3) detailed description of sample preparation protocols, including methods for organic matter digestion and PMF extraction; (4) implementation of quality assurance measures, such as recovery of internal standards (spikes); and (5) experimental replication to assess variability. Studies that did not meet these criteria (e.g., those based solely on visual identification) were considered lower-quality evidence and used cautiously, primarily to contextualize general trends, but not as the basis for central mechanistic conclusions.

For the analysis of this documentary corpus, a set of specialized tools was employed to enable both quantitative and qualitative examination. First, R Studio together with the Bibliometrix package was used for metadata processing, facilitating the calculation of fundamental metrics, such as publication trends over time, identification of the most productive authors, institutions, and journals, as well as the analysis of collaboration and co-citation patterns. Second, VOSviewer was essential for visualizing the intellectual structure of the field, allowing the construction of network maps of keyword co-occurrence to identify thematic clusters, co-authorship networks to map collaborations among researchers, and co-citation networks to recognize foundational documents and the most influential schools of thought. Finally, Plotly Studio was used to generate interactive visualizations and dynamic dashboards of the most relevant findings, thereby enhancing the exploration and dissemination of complex results derived from the analysis. This methodological triangulation provided a robust and reproducible foundation for diagnosing the state of the art, identifying knowledge gaps, and proposing future research directions in this interdisciplinary field.

### Limitations of the Search Strategy

While this review provides a comprehensive overview of the field, certain inherent limitations of the search strategy must be acknowledged. The study exclusively utilized the Scopus database. Although Scopus is widely regarded as a leading abstract and citation database for peer-reviewed literature, particularly in the environmental sciences, its sole use may introduce systematic coverage bias. Furthermore, the search was restricted to documents published in English. This language criterion, while necessary for consistency and accessibility, likely excludes significant regional scientific developments. This is particularly relevant in the context of our findings, which identified China as the dominant producer in this research area. It is plausible that a substantial body of applied research, pilot-scale studies, or local monitoring data is published in Mandarin within Chinese journals not indexed in Scopus or written in English. The omission of such regionally important work could skew the global perspective, potentially overlooking local technological innovations or specific contamination patterns. Consequently, the findings synthesized here, while robust within the indexed English-language corpus, may not capture the full heterogeneity in global research efforts. Future studies should consider multi-database approaches (e.g., incorporating Web of Science, China National Knowledge Infrastructure) and language-inclusive searches to validate and expand upon the patterns identified in this review.

## 4. Conclusions

This systematic review, informed by analysis, has mapped the current state of knowledge on the impact of microscopic plastic fibers on activated sludge dewatering, but more importantly, it has identified the main limitations and uncertainties that the scientific community must address.

- The field exhibits characteristics of a rapidly evolving research front, transitioning from descriptive occurrence studies to hypothesis-driven mechanistic inquiry. However, this quantitative expansion has not yet translated into full scientific maturity due to prevailing methodological heterogeneity.
- The data confirmed exponential growth and high productivity, dominated by China. However, this activity does not equate to consolidated scientific maturity. The field is undergoing a critical transition from the detection and quantification phase (2015–2020), based on techniques such as micro-FTIR [42,43], to the mechanistic elucidation phase (2020–present), driven by omics approaches [15]. The absence of terms such as EPSs and proteomics in the core lexicon of the most cited literature (Table 3) demonstrates that this latter strand remains an emerging subdiscipline, not yet fully integrated into mainstream discourse.
- Mechanistic advances, but with significant methodological uncertainties.

Progress has been made in understanding that PMFs—particularly polyester fibers—selectively adsorb hydrophobic EPS proteins, altering floc structure and impairing dewaterability [14]. However, these findings must be interpreted with caution due to strong methodological dependence and lack of standardization. Conclusions are intrinsically tied to the characterization techniques employed (Table 5), and variability in sampling, blank controls, and quantification methods introduces uncertainties that limit the generalization of results [3,41]. The reliability of the evidence is therefore heterogeneous, requiring critical case-by-case evaluation.

- Toward a second-generation research agenda.

Future research must move beyond simple documentation of the problem and address the limitations identified:

Methodological standardization: It is imperative to develop and adopt harmonized protocols for sampling, extraction, identification (spectroscopic rather than visual), and quantification of PMFs in sludge, as well as for dewaterability assessment. This will enable cross-study comparison and meta-analysis. Validation under real conditions: Laboratory mechanistic studies must be complemented by pilot-scale and full-scale plant validations, accounting for temporal variability and the complexity of the sludge matrix, including combined (“cocktail”) effects with other contaminants [8]. Multi-omics integration and modeling: Deeper integration of proteomics with metabolomics and functional genomics is needed to unravel the metabolic and signaling pathways affected by PMFs. In parallel, predictive models based on machine learning and molecular dynamics simulations [24] can help overcome experimental limitations and guide the design of smarter mitigation strategies.

Although bibliometric analysis reveals a vibrant field, critical synthesis of the literature shows that knowledge of the molecular mechanisms linking PMFs to sludge dewatering remains incipient and burdened by significant methodological uncertainties. Overcoming these limitations is the necessary step to transform scientific findings into robust engineering solutions that safeguard the economic and environmental sustainability of sludge management in the era of plastic pollution.

## Figures and Tables

**Table 1:** Frequency and temporal evolution of the main keywords in research.

#	Keyword	Absolute Frequency	Frequency	Residual	Standardized Residual	First Year	Last Year	Years	Annual Growth Rate (%)
1	microplastic	1111	2.578	1106.18	503.5931	2016	2026	11	41.42
2	microplastics	1044	2.4225	1039.18	473.0909	2016	2026	11	29.73
3	wastewater treatment	549	1.2739	544.18	247.7391	2007	2026	20	13.45
4	polymer	515	1.195	510.18	232.2604	2003	2026	24	12.16
5	environmental monitoring	427	0.9908	422.18	192.1978	2017	2026	10	−3.67
6	plastic	422	0.9792	417.18	189.9216	2016	2026	11	0
7	wastewater	385	0.8936	380.18	173.0771	2007	2026	20	12.88
8	sewage	351	0.8145	346.18	157.5984	2017	2026	10	−2.01
9	water pollutant	343	0.7959	338.18	153.9563	2017	2026	10	2.05
10	water treatment	331	0.7681	326.18	148.4932	2003	2026	24	10.53

Note: Includes early access publications with a formal publication date in 2026, available online at the time of the database search.

**Table 2:** Bibliometric metadata of key authors in research on plastic microfibers, sludge and EPSs.

Author	Number of Articles	Total Citations	Average Citations	H-Index	G-Index	Country	Primary Affiliation	First Year of Publication	Last Year of Publication	Annual Productivity
Wang, Y.	28	1077	38.46	17	29	China	Beijing University of Technology, Beijing, China; Beijing University of Technology	2020	2026	4
Zhang, Y.	23	1691	73.09	18	23	China	School of Ecology and Environment, North China University of Water Resources and Electric Power, Zhengzhou, China	2020	2025	3.83
Wang, H.	17	911	53.59	10	17	China	College of Environment, Zhejiang University of Technology, Hangzhou, Zhejiang, C	2016	2026	1.55
Wang, Z.	17	613	36.06	11	17	Finland	College of Energy and Environment, Inner Mongolia University of Science and Tech	2020	2025	2.83
Chen, X.	16	423	26.44	8	16	China	School of Energy and Environment, Zhongyuan University of Technology, Zhengzhou,	2021	2026	2.67
Zhang, Z.	15	992	66.13	8	15	China	College of River and Ocean Engineering, Chongqing Jiaotong University, Chongqing	2020	2025	2.5
Li, J.	15	730	48.67	8	15	China	School of Civil Engineering, Chongqing Jiaotong University, Chongqing, Chongqing	2020	2025	2.5
Li, Y.	15	432	28.8	10	15	China	School of Energy and Environment, Zhongyuan University of Technology, Zhengzhou,	2021	2025	3
Liu, Y.	13	277	21.31	7	13	China	College of Environment, Liaoning University, Shenyang, Liaoning, China	2021	2025	2.6
Vollertsen J.	12	1982	165.17	10	12	Denmark	Department of Civil Engineering, Aalborg University, Aalborg, Mitsuyi/JinXi, Denmark	2018	2025	1.5

Note: Includes early access publications with a formal publication date in 2026, available online at the time of the database search.

**Table 3:** Metadata and metrics of scientific publications on the area of study.

	Title	Number of Citations	Main Author	Year of Publication	Journal	Document Type	Citations per Year	Country of Affiliation of Main Author	Affiliation of Main Author
1	Microplastics in freshwaters and drinking water: Critical review and assessment of data quality [41].	1989	Koelmans, A.A.	2019	*Water Research*	Review	331.5	The Netherlands	Aquatic Ecology and Water Quality Management Group, Wageningen University & Research, Wageningen, Ge
2	Identification of microplastic in effluents of waste water treatment plants using focal plane array-based micro-Fourier-transform infrared imaging [42]	1275	Mintenig, S.M.	2017	*Water Research*	Article	159.38	Germany	Alfred-Wegener-Institut Helmholtz-Zentrum für Polar- und Meeresforschung, Bremerhaven, Bremen, Germany
3	Wastewater treatment plants as a pathway for microplastics: Development of a new approach to sample wastewater-based microplastics [43].	1216	Zijajahromi, S.	2017	*Water Research*	Article	152	Australia	Griffith School of Environment, Griffith University, Brisbane, QLD, Australia
4	(Nano)plastics in the environment—Sources, fates and effects [46].	964	Pinto da Costa, J.P.	2016	*Science of the Total Environment*	Review	133	China	School of Environmental and Chemical Engineering, Shanghai University, Shanghai, China
5	Microplastics in sewage sludge from the wastewater treatment plants in China [44].	931	Li, X.	2018	*Water Research*	Article	107.86	Denmark	Department of Civil Engineering, Aalborg University, Aalborg, Nordyiland, Denmark
6	Quantification of microplastic mass and removal rates at wastewater treatment plants applying Focal Plane Array (FPA)-based Fourier Transform Infrared (FT-IR) imaging [47].	755	Simon, M.	2018	*Water Research*	Article	107.11	Portugal	Universidade de Aveiro, Aveiro, Aveiro, Portugal
7	Retention of microplastics in a major secondary wastewater treatment plant in Vancouver, Canada [48].	589	Gies, E.A.	2018	*Marine Pollution Bulletin*	Article	105.2	China	State Key Laboratory of Pollution Control and Resource Reuse, Tongji University, Shanghai, China
8	Fast identification of microplastics in complex environmental samples by a thermal degradation method [49].	570	Duemichen, E.	2017	*Chemosphere*	Article	104	Spain	Department of Analytical Chemistry, Physical Chemistry and Chemical Engineering, Universidad de Alca
9	The fate of microplastics in an Italian Wastewater Treatment Plant [50].	555	Magni, S.	2019	*Science of the Total Environment*	Article	101.25	Qatar	Environmental Science Center, Qatar University, Doha, Qatar
10	Microplastic sources, formation, toxicity and remediation: a review [45].	555	Osman, A.I.	2023	*Environmental Chemistry Letters*	Review	92.5	Italy	Department of Biosciences, Università degli Studi di Milano, Milan, MI, Italy

Note: The country listed corresponds to the institutional affiliation of the first/main author at the time of publication, as recorded in the Scopus database. This does not necessarily indicate the geographic location where the study was conducted. For example, the study in Vancouver, Canada (Gies et al., 2018 [48]), is led by an author affiliated with a Chinese institution, reflecting the international nature of research collaborations.

**Table 4:** Distribution by country of the agencies funding studies on the area.

	Country	Sources of Financing
1	China	National Natural Science Foundation (272); National Key Research and Development (62); Fundamental Research Funds for the (21); China Postdoctoral Science Foundation (18); Natural Science Foundation of Beijing (17); State Key Laboratory of Pollution C (12); Basic and Applied Basic Research Fo (11); Natural Science Foundation of Jiang (11); Ministry of Education of the People (9); Priority Academic Program Development (9)
2	United States	National Natural Science Foundation (17); National Science Foundation (13); U.S. Geological Survey (11); National Institute of Environmental (6); U.S. Department of Agriculture (5); National Research Foundation of Kor (5); National Institutes of Health (3); Major Science and Technology Program (3); National Institute of Food and Agri (3); High-end Foreign Experts Recruitment (2)
3	Australia	Australian Research Council (23); National Natural Science Foundation (14); National Key Research and Development (7); China Three Gorges Corporation (4); RMIT University (3); Griffith University (3); Enterprise Ireland (2); Science Foundation Ireland (2); Zhejiang A and F University (2); Ministry of Higher Education (2)
4	Germany	Bundesministerium für Bildung und F (14); Deutsche Forschungsgemeinschaft (5); Deutsche Bundesstiftung Umwelt (4); Merck KGaA (2); Medical Research Council (2); Fundação de Amparo à Pesquisa do Es (2); Conselho Nacional de Desenvolviment (2); National Natural Science Foundation (2); Natural Science Foundation of Jiang (2); Priority Academic Program Development (2)
5	The Netherlands	National Natural Science Foundation (4); Horizon 2020 Framework Programme (2); H2020 Marie Skłodowska-Curie Action (2); Nederlandse Organisatie voor Wetens (2); Javna Agencija za Raziskovalno Deja (2); China Postdoctoral Science Foundation (2); World Health Organization (2); Norges Teknisk-Naturvitenskapelige (1); KWR Water Research Institute (1); Institute for Sustainable Process T (1)
6	Spain	Ministerio de Ciencia e Innovación (26); Ministerio de Ciencia (17); European Commission (16); Agencia Estatal de Investigación (10); Generalitat de Catalunya (8); Fundación Séneca (8); Ministerio de Economía y Competitiv (7); Generalitat Valenciana (5); European Regional Development Fund (4); Horizon 2020 Framework Programme (4)
7	United Kingdom	Natural Environment Research Council (10); National Natural Science Foundation (9); UK Research and Innovation (6); Engineering and Physical Sciences R (4); Fundação para a Ciência e a Tecnolo (4); Horizon 2020 Framework Programme (3); Global Challenges Research Fund (3); Ministry of Education (3); National Key Research and Development (3); European Commission (3)
8	Switzerland	Schweizerischer Nationalfonds zur F (5); Eidgenössische Anstalt für Wasserve (3); Natural Science Foundation of Shang (2); National Natural Science Foundation (2); Eidgenössische Technische Hochschul (2); World Health Organization (2); Háskóli Íslands (1); Bundesamt für Umwelt (1); Fundação para a Ciência e a Tecnolo (1)
9	India	Department of Science and Technology (6); Ministry of Education (5); University Grants Commission (4); Department of Biotechnology (3); Ministry of Higher Education (3); Science and Engineering Research Bo (2); U.S. Environmental Protection Agency (2); U.S. Geological Survey (2); Bryden Centre (2); UK Research and Innovation (2)
10	Italy	Ministero dell’Istruzione (12); European Commission (8); Fondazione Cariplo (7); European Regional Development Fund (4); Università degli Studi di Firenze (2); Università degli Studi di Trento (2); Center for Colloid and Surface Scie (2); RRF Foundation for Aging (2); Comisión Interministerial de Cienci (2); Agencia Estatal de Investigación (2)

**Table 5:** Comparison of analytical methods for microplastics in sludge.

Method	Size Range	Detection Limit	Advantages	Limitations	Application in Sludge	Ref.
Micro-FTIR (transmission/reflectance)	>10–20 μm	1–10 particles	Simultaneous polymer identification, chemical mapping, non-destructive	Water interference, analysis time, size limit	Morphochemical characterization of particles > 20 μm	[15]
Micro-Raman	>1 μm	1–5 particles	High spatial resolution, minimal water interference, detects small particles	Fluorescence interference, photodegradation, weak signal in dark samples	Identification of small particles (<10 μm) and nanoplastics	[23]
Pyrolysis-GC/MS	Not applicable (total mass)	0.1–5 μg (per polymer)	Mass quantification, additive identification, polymers undetectable by spectroscopy	Destructive, no morphological information, no size distinction	Mass quantification by polymer in total extracts	[24]
TED-GC/MS	Not applicable	0.5–10 μg	Direct solid sample, less preparation, rapid analysis	Lower sensitivity than Py-GC/MS, matrix effect	Rapid screening of main polymers in dried sludge	[36]
SEM-EDX	>0.1 μm	Semi-quantitative	Detailed morphology, elemental composition	Does not identify polymers, requires coating, small area	Confirmation of suspect particles, surface analysis	[42]
Nile Red staining + fluorescence microscopy	>3 μm	10–100 particles	Fast, economical, large-volume screening	False positives, no polymer identification, organic matter interference	Rapid quantification of total abundance	[44]

**Table 6:** Comparative analysis of PMF polymer types and their differential effects on sludge dewatering.

Polymer	Contact Angle (Hydrophobicity)	Surface Functional Groups	EPS Protein Adsorption	CST Increase	Proteomic Signature	Key Reference
**Polyester (PET)**	78°	Esters (-C=O, -O-)	32.5%	+45%	Esterase overexpression	[13]
**Polypropylene (PP)**	102°	C-C, C-H only	18.2%	+22%	Oxidative stress (catalases)	[13]
**Polyamide (PA/Nylon)**	62°	Amides (-CONH-)	27.8%	+31%	Protease activation	[34]
**Polystyrene (PS)**	89°	Aromatic rings	24.1%	+28%	Aromatic stress response	[39]

**Table 7:** Functional categories of affected proteins.

Category	Examples	Expression Change	Functional Consequence
Structural matrix proteins	Glycoproteins, lectins, polysaccharide-binding proteins	↓ 60%	Reduced intercellular cohesion, floc fragmentation
Adhesion/biofilm formation	Adhesins, pili-associated proteins	↓ 45%	Weakened floc structure, increased susceptibility to shear
Stress response	Chaperones (DnaK, GroEL), heat shock proteins	↑ >400%	Metabolic resource diversion toward survival
Extracellular hydrolases	Proteases, cellulases	↑ 190–280%	Autolytic matrix degradation

**Table 8:** Integrated multi-scale research framework linking molecular mechanisms, structural responses, and engineering solutions for PMF-induced sludge dewatering impairment.

Level	Objective	Research Question	Method	Connects to…
**Molecular**	Identify protein domains with PMF affinity	Which sequences/structures determine selective adsorption?	Molecular docking, surface plasmon resonance (SPR)	Hypothesis 1 (chemical specificity)
**Cellular**	Map affected metabolic pathways	How does the cell reprogram its metabolome under PMF stress?	Metatranscriptomics + metabolomics	Sharma’s proteomic signature
**Floc**	Model 3D architecture	How is water redistributed in the presence of PMFs?	Micro-CT, cryo-SEM, rheometry	Pore size alteration
**Plant**	Validate under real conditions	Do laboratory mechanisms explain plant-scale observations?	Longitudinal monitoring, machine learning	Müller’s trade-offs
**Solution**	Design rational intervention	Can we compete with PMF–protein adsorption?	Functionalized polymers, bioaugmentation	Adsorption mechanism

## Data Availability

No new data were created or analyzed in this study. Data sharing is not applicable to this article.

## Supplementary Materials

The following supporting information can be downloaded at: https://www.mdpi.com/article/10.3390/polym18060734/s1, File S1. PRISMA 2020 Checklist. Reference [53] is cited in the Supplementary Materials.
