# Supplementary material for: Global Research Landscape on Plastic Microfibers in Sludge Treatment: Proteomic Mechanisms and Biotechnological Pathways for Biomass Valorization
Source: Polymers (Basel). 2026 Mar 17;18(6):734. doi: 10.3390/polym18060734 (PMC13030239; doi:10.3390/polym18060734)
Supplement: Supplementary file 1 [file polymers-18-00734-s001.zip › polymers-4202223-supplementary.pdf]

Section and Topic	Item #	Checklist item	Location where item is reported
TITLE			
Title	1	Identify the report as a systematic review.	Title and Abstract: The paper identifies as a "Landscape" and a "systematic review".
ABSTRACT			
Abstract	2	See the PRISMA 2020 for Abstracts checklist.	Abstract: Includes a structured summary with objectives, methodology, results, and conclusions.
INTRODUCTION			
Rationale	3	Describe the rationale for the review in the context of existing knowledge.	Introduction: Describes the accumulation of plastic microfibers (PMFs) and the lack of a unified framework on molecular mechanisms.
Objectives	4	Provide an explicit statement of the objective(s) or question(s) the review addresses.	Introduction: The main objective and five specific research questions (Q1–Q5) are detailed at the end of the section.
METHODS			
Eligibility criteria	5	Specify the inclusion and exclusion criteria for the review and how studies were grouped for the syntheses.	Methodology: Defines the 2000–2025 timeframe, English language, and core topics (PMFs, sludge, EPS).
Information sources	6	Specify all databases, registers, websites, organisations, reference lists and other sources searched or consulted to identify studies. Specify the date when each source was last searched or consulted.	Methodology: The Scopus database was used; the search was conducted in late 2025.
Search strategy	7	Present the full search strategies for all databases, registers and websites, including any filters and limits used.	Methodology: Provides the complete

https://doi.org/10.3390/polym18060734

Section and Topic	Item #	Checklist item	Location where item is reported
			search equation with Boolean operators used in Scopus.
Selection process	8	Specify the methods used to decide whether a study met the inclusion criteria of the review...	Methodology: Describes a multi-step selection process illustrated in the selection flowchart (Figure 6).
Data collection process	9	Specify the methods used to collect data from reports...	Methodology: Details the use of specialized tools like Bibliometrix (R Studio), VOSviewer, and Plotly Studio.
Data items	10a	List and define all outcomes for which data were sought.	Methodology: Includes bibliometric metrics (productivity, citation) and mechanistic findings (PMF–EPS interaction).
	10b	List and define all other variables for which data were sought.	Methodology/Results: Includes author affiliation, country, funding sources, and key research terms.
Study risk of bias assessment	11	Specify the methods used to assess risk of bias in the included studies...	Methodology: A quality filter with five robustness criteria (controls, spectroscopic confirmation, etc.) was applied.
Effect measures	12	Specify for each outcome the effect measure(s)... used in the synthesis or presentation of results.	Results: Uses bibliometric metrics (R^2, growth rates) and experimental data (CST increase, % adsorption).
Synthesis	13a-	Describe the processes used to decide which studies were eligible for each synthesis... describe any methods used to synthesize	Methodology/Analysis:

Section and Topic	Item #	Checklist item	Location where item is reported
methods	f	results...	Combines quantitative bibliometric analysis and qualitative critical review; comparisons are made with related environmental fields.
Reporting bias assessment	14	Describe any methods used to assess risk of bias due to missing results...	Methodology (Limitations): Discusses potential bias arising from the use of a single database and the English language.
Certainty assessment	15	Describe any methods used to assess certainty (or confidence) in the body of evidence for an outcome.	Methodology: A methodological quality filter was used to categorize the evidence based on robustness.
RESULTS			
Study selection	16a	Describe the results of the search and selection process... ideally using a flow diagram.	Methodology/Results: 918 documents were identified and refined to 839; details are shown in Figure 6.
	16b	Cite studies that might appear to meet the inclusion criteria, but which were excluded, and explain why they were excluded.	Methodology: 79 articles were excluded due to lack of depth in EPS or a focus strictly on effluents.
Study characteristics	17	Cite each included study and present its characteristics.	Results: Characteristics are presented in tables detailing key authors, journals, countries, and highly cited foundational works.
Risk of bias in studies	18	Present assessments of risk of bias for each included study.	Analysis: Mentions studies with higher methodological robustness (e.g.,

https://doi.org/10.3390/polym18060734

Section and Topic	Item #	Checklist item	Location where item is reported
			Müller et al. 2025) versus preliminary studies.
Results of individual studies	19	For all outcomes, present, for each study: (a) summary statistics for each group and (b) an effect estimate...	Results/Analysis: Specific findings like 32.5% protein sequestration and a 45% increase in CST are cited from individual studies.
Results of syntheses	20a-d	For each synthesis, briefly summarise the characteristics... Present results of all statistical syntheses conducted.	Results: Presented in Tables 1–3 and through analysis of the exponential growth model (R^2 = 0.9786).
Reporting biases	21	Present assessments of risk of bias due to missing results... for each synthesis assessed.	Methodology (Limitations): Discussion regarding the exclusion of literature in other languages, specifically Mandarin.
Certainty of evidence	22	Present assessments of certainty (or confidence) in the body of evidence for each outcome assessed.	Analysis/Conclusions: Concludes that scientific maturity is still limited due to methodological heterogeneity and lack of standardized protocols.
DISCUSSION			
Discussion	23a-d	Provide a general interpretation of the results... Discuss any limitations... Discuss implications...	Results (Future Directions) and Conclusions: Proposes 5 strategic directions and discusses the limitations of relying on lab-scale models.
OTHER			

Section and Topic	Item #	Checklist item	Location where item is reported
INFORMATION			
Registration and protocol	24a-c	Provide registration information for the review... or state that the review was not registered.	Methodology: Indicates that registration is pending at the Open Science Framework (OSF).
Support	25	Describe sources of financial or non-financial support for the review...	Funding: Explicitly declares that the research received no external funding.
Competing interests	26	Declare any competing interests of review authors.	Conflicts of Interest: The authors declare that they have no conflicts of interest.
Availability of data, code and other materials	27	Report which of the following are publicly available and where they can be found...	Data Availability Statement: Original contributions presented in the study are included in the article.

From: Page MJ, McKenzie JE, Bossuyt PM, Boutron I, Hoffmann TC, Mulrow CD, et al. The PRISMA 2020 statement: an updated guideline for reporting systematic reviews. BMJ 2021;372:n71. doi: 10.1136/bmj.n71. This work is licensed under CC BY 4.0. To view a copy of this license, visit https://creativecommons.org/licenses/by/4.0/

Disclaimer/Publisher's Note: The statements, opinions and data contained in all publications are solely those of the individual author(s) and contributor(s) and not of MDPI and/or the editor(s). MDPI and/or the editor(s) disclaim responsibility for any injury to people or property resulting from any ideas, methods, instructions or products referred to in the content.

https://doi.org/10.3390/polym18060734
